# Effect of transforaminal endoscopic discectomy extent on postoperative neurological recovery in lumbar disc herniation: a retrospective cohort study

**DOI:** 10.3389/fneur.2025.1728213

**Published:** 2026-01-08

**Authors:** Liang Liu, Dong Li, Xinge Liu, Hao Fu, Yongcun Geng

**Affiliations:** Department of Orthopedics, Zibo Municipal Hospital, Zibo, Shandong, China

**Keywords:** lumbar disc herniation, neurological recovery, operative time, percutaneous endoscopic lumbar discectomy, retrospective cohort study

## Abstract

**Objective:**

To investigate whether limited discectomy versus aggressive disc removal influences postoperative neurological recovery in lumbar disc herniation patients undergoing transforaminal endoscopic discectomy.

**Methods:**

This retrospective cohort study analyzed 288 patients undergoing percutaneous endoscopic lumbar discectomy (PELD) at our institution between January 2022 and January 2025. Patients were stratified by surgical approach based on established criteria: Aggressive Discectomy Group (comprehensive removal of herniated disc and extensive nucleus pulposus extraction, *n* = 135) versus Limited Discectomy Group (selective neural decompression preserving disc architecture, *n* = 153). Primary outcome was neurological recovery at 6 months, assessed via MRC motor grading and sensory function testing. Secondary outcomes included visual analog scale (VAS) pain scores, Oswestry disability index (ODI), operative duration, and complications.

**Results:**

Both groups demonstrated significant improvement in neurological function from baseline to 6 months postoperatively. No significant differences emerged between groups regarding sensory function recovery (normal/reduced sensation at 6 months: 126/9 vs. 149/4, *χ*^2^ = 2.732, *p* = 0.098) or motor strength recovery. Mean VAS scores declined from 5.41 ± 1.06 to 0.40 ± 0.55 (Aggressive group) and from 5.39 ± 1.23 to 0.53 ± 0.61 (Limited group) with no significant between-group differences (*p* > 0.05). ODI scores improved from 61.96 ± 8.52 to 23.21 ± 4.53 (Aggressive group) and from 63.62 ± 7.96 to 22.63 ± 4.82 (Limited group, *p* > 0.05). However, operative duration was significantly shorter in the Limited Discectomy Group (100.41 ± 32.33 vs. 108.48 ± 31.61 min, *p* = 0.034). No infections, hematomas, nerve root injuries, or recurrences occurred in either group during follow-up.

**Conclusion:**

Limited discectomy achieved equivalent neurological recovery outcomes compared to aggressive disc removal while requiring significantly less operative time. These findings support adopting less extensive surgical approaches when adequate neural decompression can be accomplished, potentially reducing surgical trauma while maintaining therapeutic efficacy. While these 6-month findings support limited discectomy for early recovery, longer follow-up studies are needed to assess medium to long-term outcomes including recurrence rates and degenerative changes.

## 1 Introduction

Lumbar disc herniation (LDH) constitutes the predominant spinal disorder responsible for workforce disability, with symptomatic disc herniation affecting approximately 5.5% of the worldwide population (1). Low back pain—a primary manifestation of LDH—affected 619 million people globally in 2020, projected to reach 843 million by 2050, predominantly impacting working-age adults at the L4-5 level (2). China harbors over 200 million patients with lumbar spine disorders, representing a substantial public health burden (3). The incidence among young adults (25–39 years) reaches 13.93%, representing the highest prevalence across all age groups. With widespread adoption of sedentary lifestyles, the number of LDH patients in China demonstrates an annually increasing trend, imposing tremendous pressure on healthcare resources (4). In such a clinical context, for intractable LDH, surgery is often ultimately required; however, in most cases, conservative management options may appropriately delay surgical intervention (5, 6). Following surgical intervention, patients face significant challenges with recurrence rates ranging from 0.5% to 21% and reoperation rates of 5.2%–19% (7).

Transforaminal endoscopic discectomy represents remarkable advancement in minimally invasive treatment for LDH (8). This technique accesses the surgical site through the natural anatomical pathway—the intervertebral foramen—facilitating precise operations under direct visualization while preserving posterior spinal structure integrity (9). Compared to conventional open surgery, transforaminal endoscopy demonstrates substantial advantages: surgical incisions less than 1 centimeter, 86% reduction in intraoperative bleeding, 65% decrease in hospitalization duration, immediate postoperative mobilization, and markedly reduced tissue damage and scar formation (10). Multi-center randomized controlled studies confirm that transforaminal endoscopy achieves comparable symptom relief to open surgery while demonstrating superior performance in recovery speed, complication rates, and patient satisfaction (11). This technique has rapidly gained popularity in China, becoming one of the mainstream treatment options for LDH (12).

However, controversy persists regarding the optimal extent of resection during transforaminal endoscopic surgery (13). Current clinical practice encompasses two principal strategies: limited discectomy focused on neural decompression exclusively, or aggressive discectomy involving comprehensive resection of herniated disc material and extensive nucleus pulposus removal. Advocates for aggressive discectomy argue that thorough removal of pathological tissue reduces recurrence risk (14). Conversely, research supporting limited discectomy emphasizes that excessive resection may compromise disc biomechanical structure, increase surgical trauma and nerve root injury risk, leading to long-term spinal instability (15). Systematic reviews reveal that existing studies primarily focus on comparisons between different approach methods, while direct comparative studies examining the relationship between resection extent and neurological recovery remain limited (16).

This study aims to directly compare the effects of limited versus aggressive discectomy strategies on neurological recovery through retrospective cohort analysis. We hypothesize that different resection extents demonstrate no substantial difference in neurological recovery outcomes, providing evidence-based support for selecting less traumatic surgical strategies.

## 2 Methods

### 2.1 Study population

This retrospective cohort study included patients with lumbar disc herniation who underwent lateral approach percutaneous endoscopic lumbar discectomy (PELD) at Zibo Municipal Hospital, Shandong Province, China, from January 2022 to January 2025. Sample size was determined based on study feasibility, with 288 consecutive patients meeting inclusion criteria enrolled during the study period, followed until July 2025. The diagnosis of lumbar disc herniation was established based on clinical symptoms (low back and leg pain, nerve root irritation signs) combined with imaging examinations. Magnetic resonance imaging (MRI) diagnostic criteria included posterior disc herniation with nerve root compression signs, encompassing nerve root displacement and dural sac compression deformity.

Sample size calculation was based on 80% statistical power to detect a 10% difference in neurological recovery rate between groups, assuming a significance level of *α* = 0.05. Considering a possible 10% loss rate, at least 130 patients were needed in each group. Our final sample (135 in the extensive discectomy group and 153 in the limited discectomy group) met this requirement.

The choice of surgical approach (limited versus aggressive discectomy) was based on the surgeon’s clinical judgment and not predetermined by study protocol, reflecting real-world clinical practice patterns.

Inclusion criteria: (1) adult patients (≥18 years) with single-level lumbar disc herniation (L3/4, L4/5, or L5/S1) confirmed by MRI; (2) clinical presentation consistent with radiculopathy including leg pain distribution corresponding to the affected nerve root, positive straight leg raise test, and neurological deficits; (3) failure of standardized conservative treatment for minimum 6–8 weeks including physical therapy, nonsteroidal anti-inflammatory drugs, and epidural steroid injections; (4) surgical treatment with PELD via transforaminal approach; (5) complete medical records documenting surgical technique and extent of discectomy; (6) minimum 6-month postoperative follow-up with complete neurological assessment data.

Exclusion criteria: (1) previous lumbar spine surgery at any level; (2) multi-level disc herniation requiring simultaneous surgical intervention; (3) concomitant spinal pathology including spinal stenosis, spondylolisthesis ≥Meyerding Grade I, spinal deformity with Cobb angle >10°, infection, or neoplasm; (4) severe medical comorbidities significantly impacting surgical outcomes or recovery; (5) neurological or psychiatric disorders precluding reliable outcome assessment; (6) incomplete surgical documentation preventing accurate classification of discectomy extent; (7) loss to follow-up before 6 months postoperatively.

**Figure 1:**
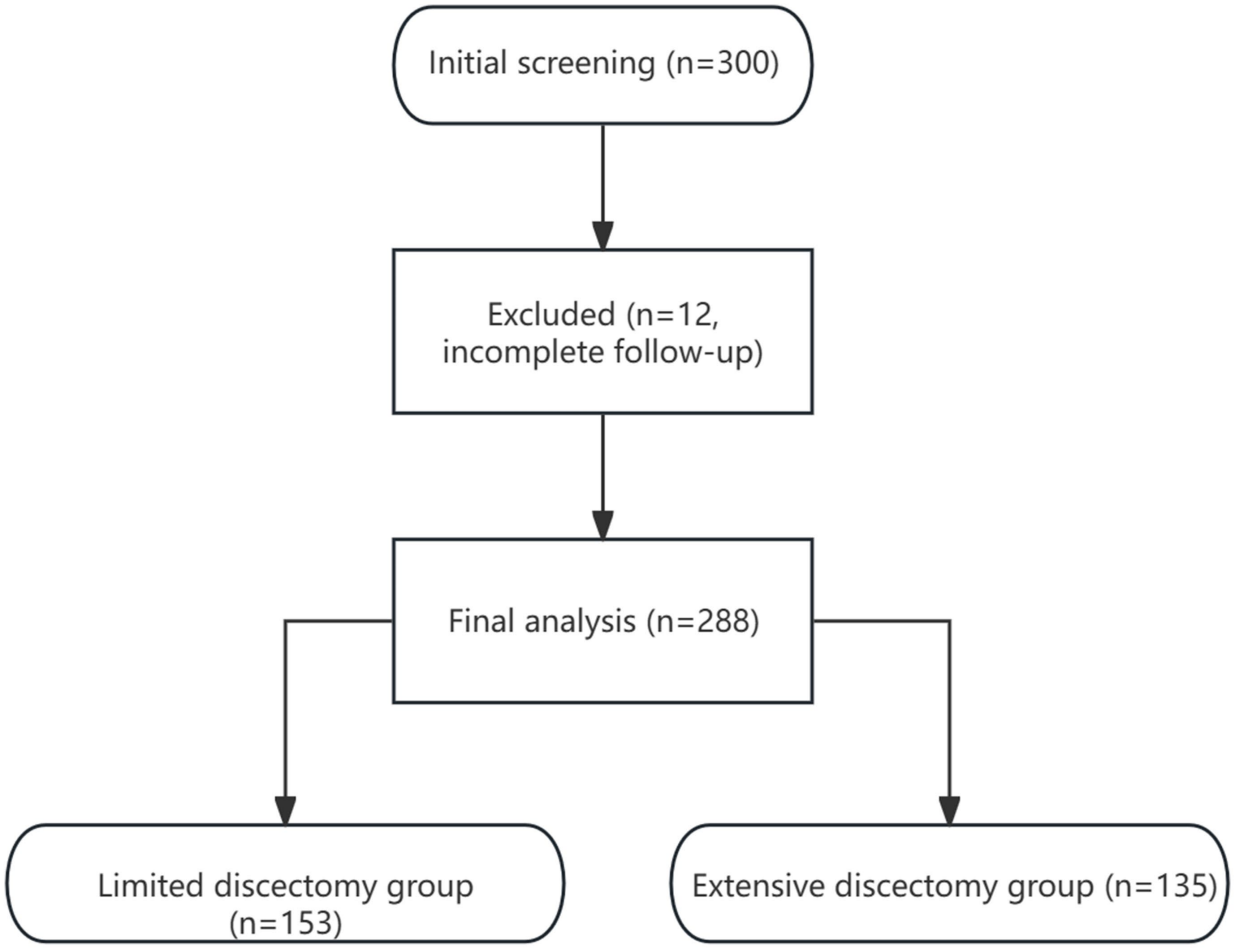
STROBE flow diagram of study population. Flow diagram showing patient screening, exclusion, and group allocation.

From the initial 300 patients, 12 were excluded due to incomplete follow-up data, resulting in a final study cohort of 288 patients (Figure 1).

### 2.2 Variables

The exposure variable was defined as the extent of neural decompression during lateral approach PELD surgery, categorized based on surgical records into Limited Discectomy Group (selective neural decompression preserving disc architecture) and Aggressive Discectomy Group (comprehensive removal of herniated disc and extensive nucleus pulposus extraction). Patient allocation to either surgical approach was determined by the attending surgeon’s clinical decision-making, primarily based on: (1) severity of clinical presentation; (2) degree and type of disc herniation observed on imaging studies; (3) patient age and comorbidities; and (4) surgeon’s expertise and preference. Specifically, aggressive discectomy was generally preferred for severe, massive, or free herniated disc material, while limited discectomy was typically selected for mild to moderate, contained herniations without significant free fragments. We acknowledge the potential for selection bias with this allocation method and therefore conducted detailed baseline characteristic comparisons to ensure no significant differences existed between groups regarding demographics and preoperative clinical parameters.

The primary outcome variable was defined as neurological recovery at 6 months postoperatively, assessed based on motor strength evaluation [Medical Research Council (MRC) motor grading scale 0–5] (17) and sensory function examination (pinprick and light touch sensation categorized as normal/reduced/absent) (18). The MRC scale represents the most widely accepted method of grading muscle strength clinically (19).

Covariates included Visual analog scale (VAS, 0–10 points) (20) and Oswestry disability index (ODI, 0–100%) (21), collected through standardized questionnaires within 1 week preoperatively, within 1 week postoperatively, and at 1 month and 6 months postoperatively. The VAS represents the patient-reported outcome measure most frequently used to measure pain intensity in low back pain trials (20). The ODI serves as a patient-completed questionnaire providing a subjective percentage score of functional disability, showing superior predictive value compared to VAS for lumbar spine motion parameters (22). Additional covariates encompassed herniation level, operative time, and hospital stay.

### 2.3 Surgical technique classification

#### 2.3.1 Limited discectomy group (tissue-preserving approach)

**Figure 2:**
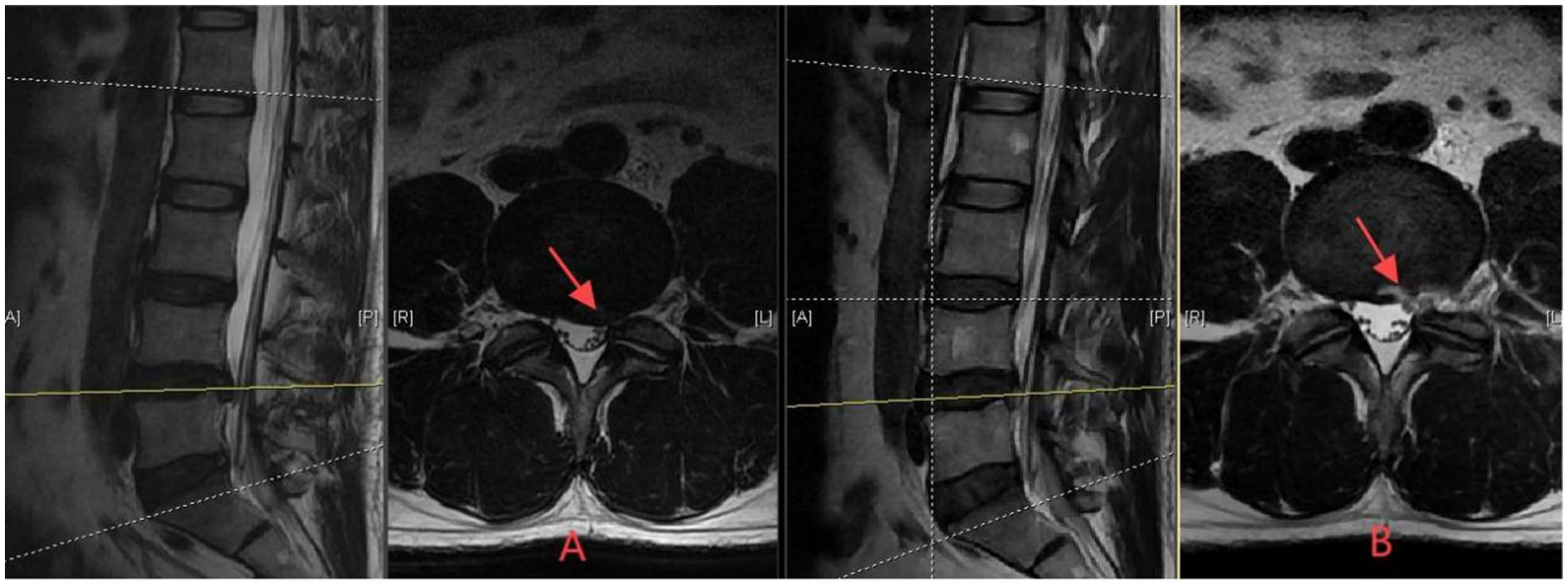
Axial T2-weighted MRI demonstrating limited discectomy approach for L4/5 disc herniation. **(A)** Preoperative image showing posterolateral disc herniation with nerve root compression (red arrow). **(B)** One-week postoperative image following selective extraction of herniated fragments causing neural compression, without entry into intact disc space. Note maintained disc architecture (red arrow indicates decompressed neural elements), demonstrating the tissue-preserving surgical strategy with neurological improvement.

This approach emphasizes neural decompression while maximizing preservation of disc structural integrity:

- Targeted fragment removal: Selective extraction strictly limited to visibly herniated disc fragments directly causing neural compression.
- Preservation principle: deliberate avoidance of entering the intact central disc space beyond the herniation site.
- Procedural endpoint: surgery terminated immediately upon achieving free mobilization of the compressed neural element, verified by direct visualization of nerve root pulsation.
- Minimal tissue disruption: preservation of annulus fibrosus continuity wherever technically feasible.
- Selective ligament resection: removal restricted to hypertrophic ligamentum flavum portions directly compressing neural structures.
- Conservative bony resection: minimal superior articular process modification preserving joint integrity (Figure 2).

#### 2.3.2 Aggressive discectomy group (comprehensive removal approach)

**Figure 3:**
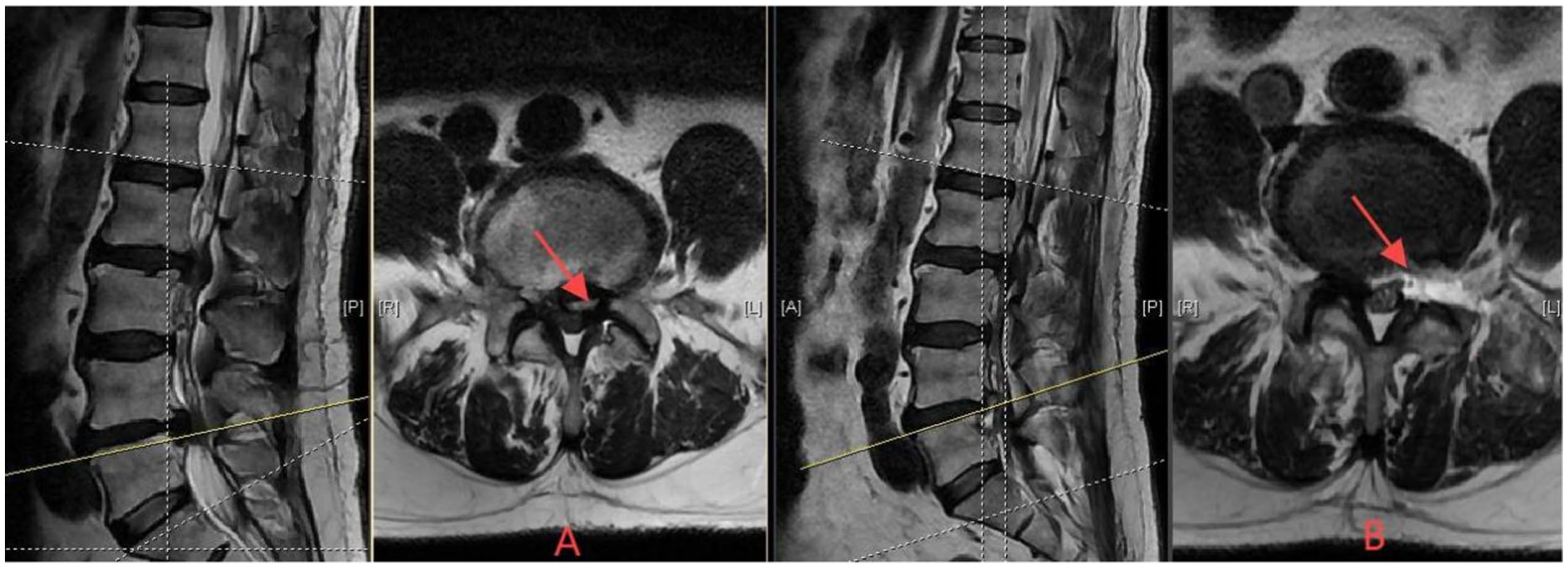
Axial T2-weighted MRI demonstrating aggressive discectomy approach for L4/5 disc herniation. **(A)** Preoperative image showing posterolateral disc herniation with nerve root compression (red arrow). **(B)** One-week postoperative image following comprehensive removal of herniated disc and extensive nucleus pulposus extraction with systematic disc space exploration. Note extensive tissue removal and enlarged disc space (red arrow indicates decompressed area), demonstrating the comprehensive removal surgical strategy with neurological improvement.

This approach involves thorough disc space exploration and extensive tissue removal:

- Systematic disc space exploration: comprehensive entry into the intervertebral disc space beyond the herniation site.
- Extended removal protocol: thorough extraction of nucleus pulposus material from central, ipsilateral, contralateral, and cranio-caudal regions of the disc space.
- Prophylactic approach: removal of all potentially mobile disc fragments, regardless of their current impact on neural structures.
- Procedural endpoint: surgery terminated only after thorough curettage confirms no additional loose disc material remains.
- Extended bony resection: more aggressive superior articular process resection to facilitate comprehensive access to central, ipsilateral and contralateral disc regions. This approach represents one end of the spectrum of discectomy techniques that prioritizes complete visualization and access over facet preservation (Figure 3).

#### 2.3.3 Intraoperative differentiation between techniques

The primary distinction between limited and aggressive approaches was operationalized through:

- Surgical intent and scope: in limited discectomy, surgeons intentionally restricted removal to only the herniated fragment, while in aggressive discectomy, surgeons systematically explored and cleared the disc space.
- Instrument utilization pattern: limited discectomy primarily employed grasping forceps for direct fragment extraction, while aggressive discectomy additionally used curettes and rongeurs for systematic disc space clearance.
- Anatomical boundary respect: limited discectomy preserved the natural boundary between herniated fragment and intact disc, while aggressive discectomy intentionally crossed this boundary to access deeper disc material.

### 2.4 Standardized surgical procedure

All procedures were performed by surgeons with extensive endoscopic experience (>10 years) using standardized PELD technique. Patients were positioned laterally under local anesthesia with intravenous sedation. Using fluoroscopic guidance, a posterolateral transforaminal approach was employed with 16-gauge puncture needle placement, sequential dilation, and 8 mm working cannula insertion. Decompression was performed according to predetermined surgical approach, followed by hemostasis and layered closure.

### 2.5 Statistical analysis

Statistical analysis was performed utilizing SPSS 24.0. Continuous variables were expressed as mean ± standard deviation. Repeated measures analysis of variance (ANOVA) was utilized to compare VAS scores and ODI scores between groups, with Greenhouse–Geisser correction applied when sphericity assumption was violated. Independent samples *t*-tests were used for between-group comparisons. Categorical variables were analyzed using *χ*^2^ tests. A significance level of *α* = 0.05 was established.

### 2.6 Blinding design

To reduce assessment bias, postoperative neurological function assessment was conducted by two independent neurosurgeons who were unaware of the patients’ surgical groups. VAS and ODI scores were completed by patients themselves, unaffected by assessors. After data collection was completed, data analysis was performed by a statistician who did not participate in clinical assessment.

### 2.7 Ethics statement

This study was approved by the Medical Ethics Committee of Zibo Municipal Hospital (Approval number: 20250917). Due to the retrospective design utilizing anonymized pre-existing clinical data, the Ethics Committee granted a waiver of informed consent. The study strictly adhered to the ethical principles of the Declaration of Helsinki.

## 3 Results

### 3.1 Patient demographics and baseline characteristics

This retrospective cohort study analyzed 288 patients who underwent percutaneous endoscopic lumbar discectomy (PELD), stratified by surgical approach: Aggressive Discectomy Group (comprehensive removal of herniated disc and extensive nucleus pulposus extraction, *n* = 135) and Limited Discectomy Group (selective neural decompression preserving disc architecture, *n* = 153). Comprehensive baseline comparisons demonstrated no statistically significant differences between groups regarding demographics, comorbidities, or preoperative clinical parameters (*p* > 0.05). However, operative time showed a statistically significant difference, with the Limited Discectomy Group requiring shorter surgical duration (100.41 ± 32.33 vs. 108.48 ± 31.61 min, *p* = 0.034) (Table 1).

**Table 1:** Baseline patient characteristics and demographic comparison.

Variable	Aggressive discectomy group (*n* = 135)	Limited discectomy group (*n* = 153)	Statistic	*p*-value
Gender			*χ*^2^ = 1.039	0.308
Male	69 (51.11%)	69 (45.10%)		
Female	66 (48.89%)	84 (54.90%)		
Age (years)	60.58 ± 13.90	60.86 ± 14.21	*t* = −0.168	0.867
Hospital stay (days)	6.66 ± 1.70	6.59 ± 2.08	*t* = 0.285	0.776
Herniation level			*χ*^2^ = 5.758	0.056
L3/4	28 (20.74%)	24 (15.69%)		
L4/5	93 (68.89%)	98 (64.05%)		
L5/S1	14 (10.37%)	31 (20.26%)		
Operative time (minutes)	108.48 ± 31.61	100.41 ± 32.33	*t* = 2.136	**0.034**
Hypertension			*χ*^2^ = 1.295	0.255
Absent	95 (70.37%)	98 (64.05%)		
Present	40 (29.63%)	55 (35.95%)		
Diabetes mellitus			*χ*^2^ = 1.665	0.197
Absent	114 (84.44%)	137 (89.54%)		
Present	21 (15.56%)	16 (10.46%)		
Smoking status			*χ*^2^ = 0.062	0.804
Non-smoker	116 (85.93%)	133 (86.93%)		
Smoker	19 (14.07%)	20 (13.07%)		
Preoperative sensory function			*χ*^2^ = 2.356	0.125
Normal	61 (45.19%)	83 (54.25%)		
Reduced	74 (54.81%)	70 (45.75%)		
Preoperative great toe dorsiflexion (MRC)			*χ*^2^ = 5.108	0.276
Grade 0	5 (3.70%)	5 (3.27%)		
Grade 1	0 (0.00%)	0 (0.00%)		
Grade 2	7 (5.19%)	5 (3.27%)		
Grade 3	20 (14.81%)	15 (9.80%)		
Grade 4	30 (22.22%)	50 (32.68%)		
Grade 5	73 (54.07%)	78 (50.98%)		
Preoperative VAS score	5.41 ± 1.06	5.39 ± 1.23	*t* = 0.160	0.873
Preoperative ODI score	61.96 ± 8.52	63.62 ± 7.96	*t* = −1.709	0.089
Gender			*χ*^2^ = 1.039	0.308

***Statistically significant (*p* < 0.05). Data presented as mean ± standard deviation or frequency (percentage). VAS, visual analog scale; ODI, Oswestry disability index; MRC, medical research council motor grading scale. Values in bold indicate statistically significant differences (*p* < 0.05).

### 3.2 Neurological function recovery assessment

Chi-square analysis revealed no occurrence of surgical complications including infections, hematomas, nerve root sheath injuries, or symptom recurrence in either group during the follow-up period. Between-group comparisons of sensory function (pinprick and light touch sensation) and great toe dorsiflexion strength demonstrated no statistically significant differences at preoperative, 1-week, 1-month, and 6-month postoperative timepoints (*p* > 0.05), indicating equivalent neurological outcomes between surgical approaches across all assessment periods.

Within-group analysis revealed significant neurological recovery patterns. In the Aggressive Discectomy Group, sensory function showed statistically significant differences across the four timepoints (*χ*^2^ = 91.358, *p* < 0.001), with post-hoc pairwise comparisons demonstrating that 1-week postoperative reduced sensation differed significantly from preoperative and subsequent postoperative timepoints (*p* < 0.05). Great toe dorsiflexion strength demonstrated statistically significant improvement from preoperative to all postoperative assessments (*χ*^2^ = 68.516, *p* < 0.001).

Similarly, the Limited Discectomy Group exhibited statistically significant sensory function improvement across timepoints (*χ*^2^ = 92.789, *p* < 0.001), with 1-week postoperative reduced sensation differing significantly from preoperative and later postoperative periods (*p* < 0.05). Motor strength recovery followed an identical pattern of significant improvement from preoperative baseline (*χ*^2^ = 52.484, *p* < 0.001) (Table 2).

**Table 2:** Neurological function recovery: between-group and within-group comparisons with pairwise analysis.

Indicator	Aggressive discectomy group (*n* = 135)	Limited discectomy group (*n* = 153)	*χ*^2^ value	*p*-value
Sensory function (pinprick and light touch)				
Preoperative (normal/reduced)	61^a^/74^b^	83^a^/70^b^	2.356	0.125
1 week postoperative (normal/reduced)	93^a^/42^a^	114^a^/39^a^	1.121	0.290
1 month postoperative (normal/reduced)	114^a^/21^b^	133^a^/20^b^	0.362	0.547
6 months postoperative (normal/reduced)	126^a^/9^b^	149^a^/4^b^	2.732	0.098
Within-group *χ*^2^	91.358	92.789		
Within-group *p*	<0.001***	<0.001***		
Great toe dorsiflexion strength (MRC grade 0–5)				
Preoperative (0/1/2/3/4/5)	5^a^/0/7^a^/20^a^/30^a^/73^a^	5^a^/0/5^a^/15^a^/50^a^/78^a^	5.108	0.276
1 week postoperative (0/1/2/3/4/5)	5^a^/0/2^a,b^/5^b^/25^a^/98^b^	2^a^/0/1^a^/8^a^/39^a^/103^a^	4.390	0.356
1 month postoperative (0/1/2/3/4/5)	0^a^/0/5^a,b^/2^b^/21^a^/107^b,c^	2^a^/0/1^a^/7^a^/29^a^/114^a^	7.852	0.097
6 months postoperative (0/1/2/3/4/5)	0^a^/0/0^b^/1^b^/15^a^/119^c^	0^a,b^/0/1^a,b^/5^a,b^/15^b^/132^a^	3.228	0.358
Within-group *χ*^2^	68.516	52.484		
Within-group *p*	<0.001***	<0.001***		

***Statistically significant (*p* < 0.001). Superscript letters (a, b, c, d) indicate pairwise comparisons: identical letters indicate no statistical significance between groups, different letters indicate statistical significance between groups.

### 3.3 Pain and functional assessment (VAS and ODI scores)

Both surgical approaches achieved substantial clinical improvement with comprehensive 6-month follow-up. Within-group comparisons demonstrated statistically significant differences in VAS and ODI scores across all preoperative and postoperative timepoints (*p* < 0.05), with significant pairwise differences between any two timepoints for VAS scores, indicating progressive symptom improvement.

Between-group comparisons revealed no statistically significant differences in VAS or ODI scores at preoperative or any postoperative timepoints (*p* > 0.05). Pain intensity (VAS) improved dramatically from 5.41 ± 1.06 to 0.40 ± 0.55 in the Aggressive Discectomy Group and from 5.39 ± 1.23 to 0.53 ± 0.61 in the Limited Discectomy Group. Functional disability (ODI) demonstrated comparable improvement from 61.96 ± 8.52 to 23.21 ± 4.53 in the Aggressive Discectomy Group and from 63.62 ± 7.96 to 22.63 ± 4.82 in the Limited Discectomy Group (Table 3).

**Table 3:** Pain intensity and functional disability: longitudinal comparison between groups with pairwise analysis.

Observation variables	Control group-complete resection (*n* = 135)	Experimental group-limited decompression (*n* = 153)	*t*	95% CI	*p*-value
VAS score					
Preoperative	5.41 ± 1.06^bcd^	5.39 ± 1.23^bcd^	0.160	MD = 0.022 (−0.246, 0.289)	0.873
1 week postoperative	2.25 ± 0.84^acd^	2.20 ± 0.93^acd^	0.530	MD = 0.056 (−0.152, 0.263)	0.597
1 month postoperative	0.89 ± 0.70^abd^	0.85 ± 0.63^abd^	−0.503	MD = 0.040 (−0.114, 0.193)	0.616
6 months postoperative	0.40 ± 0.55^abc^	0.53 ± 0.61^abc^	−1.887	MD = −0.129 (−0.264, 0.006)	0.060
*F*	1045.242	961.194			
*p*-value	<0.001***	<0.001***			
ODI score					
Preoperative	61.96 ± 8.52^bcd^	63.62 ± 7.96^bcd^	−1.706	MD = −1.658 (−3.571, 0.255)	0.089
1 week postoperative	37.88 ± 6.24^acd^	37.91 ± 6.51^acd^	−0.036	MD = −0.027 (−1.512, 1.458)	0.971
1 month postoperative	30.23 ± 5.13^abd^	29.77 ± 4.64^abd^	0.808	MD = 0.465 (−0.668, 1.598)	0.420
6 months postoperative	23.21 ± 4.53^abc^	22.63 ± 4.82^abc^	1.049	MD = 0.580 (−0.509, 1.668)	0.295
*F*	969.467	1299.880			
*p*-value	<0.001***	<0.001***			

***Statistically significant (*p* < 0.001). Data presented as mean ± standard deviation. VAS, visual analog scale (0–10); ODI, Oswestry disability index (0–100%); CI, confidence interval.

Superscript notation for pairwise comparisons:

a Statistically significant difference compared to preoperative.

b Statistically significant difference compared to 1 week postoperative.

c Statistically significant difference compared to 1 month postoperative.

d Statistically significant difference compared to 6 months postoperative.

Identical superscript letters indicate no statistical significance between timepoints; different letters indicate statistical significance between timepoints.

### 3.4 Repeated measures analysis of variance

Comprehensive repeated measures ANOVA was conducted to evaluate temporal patterns and between-group differences in primary outcome measures.

VAS score analysis:

- Within-subject effect: *F* = 2578.083, *p* < 0.001, indicating statistically significant differences across temporal measurement points.
- Between-group effect: *F* = 0.002, *p* = 0.961, demonstrating no statistically significant difference between Aggressive and Limited Discectomy groups.
- Interaction effect: *F* = 0.938, *p* = 0.397, indicating no evidence of differential VAS score recovery patterns between surgical methods across timepoints.

**Figure 4:**
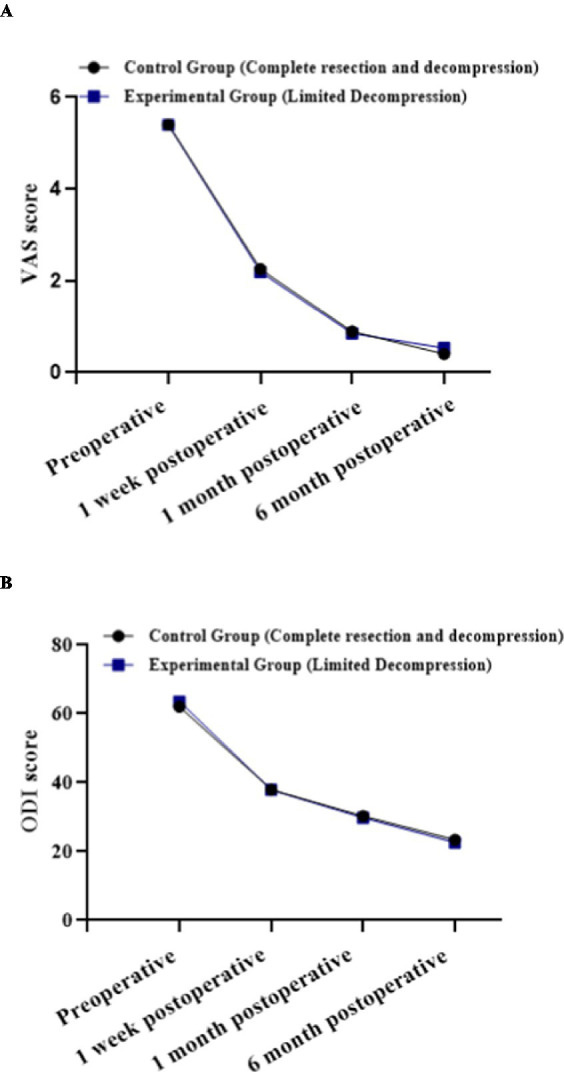
Longitudinal recovery patterns for pain intensity and functional disability. **(A)** VAS score recovery trajectory. **(B)** ODI score recovery trajectory.

ODI score analysis:

- Within-subject effect: *F* = 3407.308, *p* < 0.001, indicating statistically significant differences across temporal measurement points.
- Between-group effect: *F* = 0.094, *p* = 0.759, demonstrating no statistically significant difference between Aggressive and Limited Discectomy groups.
- Interaction effect: *F* = 3.010, *p* = 0.062, indicating no evidence of differential ODI score recovery patterns between surgical methods across timepoints (Figure 4).

VAS scores and ODI scores demonstrated identical recovery patterns between groups, with no significant between-group differences (*p* > 0.05) but highly significant within-group improvements over time (*p* < 0.001). Both surgical approaches achieved clinically meaningful improvement thresholds: VAS reduction >2 points and ODI reduction >15 points.

### 3.5 Complication profile and safety assessment

Both surgical approaches demonstrated excellent safety profiles with zero incidence of major complications during the 6-month follow-up period. No cases of surgical site infection, postoperative hematoma formation, nerve root injury, or symptom recurrence were documented in either group. The absence of complications in both groups precluded statistical comparison of safety outcomes, but this finding supports the overall safety and efficacy of both surgical approaches when performed by experienced endoscopic surgeons.

The significantly reduced operative time in the Limited Discectomy Group (8.07 min reduction, representing 7.4% efficiency gain, *p* = 0.034) may contribute to decreased anesthesia exposure, reduced radiation exposure, and potentially lower perioperative risk profiles, although no complications were observed in either group to validate this theoretical advantage.

## 4 Discussion

### 4.1 Principal findings and clinical implications

This retrospective cohort study demonstrates that limited discectomy achieves neurological recovery outcomes equivalent to aggressive disc removal in transforaminal endoscopic surgery for lumbar disc herniation. The key finding of comparable sensory function recovery (97.39% vs. 93.33% normal sensation at 6 months, *p* = 0.098) and motor strength restoration (>95% achieving MRC grades 4–5 in both groups) suggests that neural decompression efficacy may not be proportional to the volume of disc material removed. However, these findings are from a single-center retrospective study with specific inclusion criteria, limiting their generalizability to different patient populations or surgical techniques.

These findings may have several clinical implications. First, surgeons might consider prioritizing targeted decompression over extensive tissue removal when planning PELD procedures for single-level disc herniation. Second, the reduced operative time associated with limited discectomy could potentially benefit resource allocation in high-volume spine centers. Third, preservation of disc architecture might theoretically reduce long-term biomechanical alterations, though longer follow-up studies are needed to validate this hypothesis.

The statistically significant reduction in operative time (8.07 min, *p* = 0.034) observed with the limited approach, while modest in absolute terms, represents a 7.4% efficiency gain. This improvement may translate to reduced radiation exposure, decreased anesthesia requirements, and potential cost savings when applied across large patient populations.

### 4.2 Comparison with existing literature

The findings demonstrate remarkable consistency with several international investigations regarding neurological recovery outcomes following transforaminal endoscopic surgery (23). The Limited Discectomy Group achieved VAS score improvement to 0.53 ± 0.61 points at 6 months postoperatively, aligning closely with recent reports on PELD treatment for lumbar disc herniation (24). Similarly, our inter-group ODI score improvements correspond with results from Reheman et al.’s (25) retrospective cohort study, which compared clinical outcomes between limited discectomy and aggressive discectomy utilizing percutaneous endoscopic transforaminal discectomy, finding no substantial differences in VAS and ODI score improvements between groups at long-term follow-up (*p* > 0.05).

However, our study presents unique advantages in early neurological recovery assessment. Previous evidence-based reviews have demonstrated differential recovery patterns between conservative and aggressive discectomy approaches, with some studies showing significant differences in early postoperative outcomes (26). This contrasts with our finding of comparable functional recovery between both groups at 1 month postoperatively. The discrepancy may stem from different population selection criteria—our study strictly focused on single-segment posterolateral disc herniation, while broader investigations included mixed patient populations without herniation type stratification (27).

### 4.3 Biomechanical and structural considerations

From a surgical strategy perspective, the limited discectomy approach, through maximizing preservation of annulus fibrosus integrity and maintaining anterior-middle column structural stability, may reduce postoperative disc height loss and structural changes. Biomechanical studies demonstrate that endoscopic discectomy with minimal disc removal preserves spinal stability and disc height better than extensive discectomy, potentially reducing long-term degenerative changes (28, 29).

The extent of facet joint resection represents an important technical consideration in endoscopic discectomy. While more aggressive resection as observed in our Aggressive Discectomy Group facilitates comprehensive access, our findings suggest that limited discectomy with preservation of facet joints achieved equivalent neurological recovery with shorter operative times. This supports the concept that facet preservation may be preferable when adequate decompression can be achieved through less invasive means, potentially avoiding long-term biomechanical consequences associated with extensive posterior element resection.

Future research should explore optimal resection strategies for different types of disc herniations, extend follow-up periods to assess long-term recurrence patterns, and conduct multicenter prospective randomized controlled trials to validate these findings across diverse patient populations and surgical settings (30).

### 4.4 Study strengths and limitations

This study demonstrates several methodological strengths including standardized data extraction protocols, adequate sample size with well-balanced baseline characteristics, and utilization of internationally validated functional evaluation scales (VAS, ODI), ensuring clinical significance and international comparability of results (20, 21).

Several limitations should be considered when interpreting these findings. The single-center design may limit generalizability to different surgical techniques, patient populations, or healthcare systems. The 6-month follow-up period, while appropriate for assessing immediate neurological recovery, is a significant limitation of this study as it is insufficient to detect medium to long-term differences in structural outcomes or recurrence patterns. Specifically, the risk of recurrent disc herniation in limited discectomy and the development of spinal degeneration after aggressive disc removal typically manifest beyond the 6-month window, potentially between 1 and 5 years postoperatively. This limitation necessitates cautious interpretation of our findings, particularly when making recommendations about optimal surgical approaches. Additionally, as patients were not randomly assigned to treatment groups but rather underwent procedures based on surgeon’s clinical judgment, the potential for selection bias exists. However, the well-balanced baseline characteristics between groups mitigate this concern to some extent.

## 5 Conclusion

In conclusion, limited discectomy matched aggressive discectomy for neurological recovery while requiring shorter operative time in this retrospective cohort of patients with single-level lumbar disc herniation. These observations suggest that targeted neural decompression may be sufficient when technically feasible. These findings support the adoption of tissue-preserving surgical strategies, including preservation of facet joint integrity when possible. Future multicenter prospective investigations with extended follow-up periods of at least 2–5 years are essential to properly evaluate the medium to long-term outcomes, particularly regarding recurrence rates after limited discectomy and degenerative changes following aggressive disc removal. Until such data are available, clinicians should exercise caution when selecting surgical approaches based solely on our short-term findings. Randomized controlled trials with longer follow-up periods are needed to establish definitive recommendations for surgical approach selection across diverse patient populations.

## Data Availability

The original contributions presented in the study are included in the article/supplementary material, further inquiries can be directed to the corresponding author.
